# Depth-sensitive diffuse speckle contrast topography for high-density mapping of cerebral blood flow in rodents

**DOI:** 10.1117/1.NPh.10.4.045007

**Published:** 2023-11-14

**Authors:** Mehrana Mohtasebi, Dara Singh, Xuhui Liu, Faraneh Fathi, Samaneh Rabienia Haratbar, Kathryn E. Saatman, Lei Chen, Guoqiang Yu

**Affiliations:** a University of Kentucky, Department of Biomedical Engineering, Lexington, Kentucky, United States; b University of Kentucky, Spinal Cord and Brain Injury Research Center, Department of Physiology, Lexington, Kentucky, United States

**Keywords:** neuroimaging, cerebral blood flow, speckle contrast diffuse correlation topography, diffuse speckle contrast topography, parallel computation

## Abstract

**Significance:**

Frequent assessment of cerebral blood flow (CBF) is crucial for the diagnosis and management of cerebral vascular diseases. In contrast to large and expensive imaging modalities, such as nuclear medicine and magnetic resonance imaging, optical imaging techniques are portable and inexpensive tools for continuous measurements of cerebral hemodynamics. The recent development of an innovative noncontact speckle contrast diffuse correlation tomography (scDCT) enables three-dimensional (3D) imaging of CBF distributions. However, scDCT requires complex and time-consuming 3D reconstruction, which limits its ability to achieve high spatial resolution without sacrificing temporal resolution and computational efficiency.

**Aim:**

We investigate a new diffuse speckle contrast topography (DSCT) method with parallel computation for analyzing scDCT data to achieve fast and high-density two-dimensional (2D) mapping of CBF distributions at different depths without the need for 3D reconstruction.

**Approach:**

A new moving window method was adapted to improve the sampling rate of DSCT. A fast computation method utilizing MATLAB functions in the Image Processing Toolbox™ and Parallel Computing Toolbox™ was developed to rapidly generate high-density CBF maps. The new DSCT method was tested for spatial resolution and depth sensitivity in head-simulating layered phantoms and *in-vivo* rodent models.

**Results:**

DSCT enables 2D mapping of the particle flow in the phantom at different depths through the top layer with varied thicknesses. Both DSCT and scDCT enable the detection of global and regional CBF changes in deep brains of adult rats. However, DSCT achieves fast and high-density 2D mapping of CBF distributions at different depths without the need for complex and time-consuming 3D reconstruction.

**Conclusions:**

The depth-sensitive DSCT method has the potential to be used as a noninvasive, noncontact, fast, high resolution, portable, and inexpensive brain imager for basic neuroscience research in small animal models and for translational studies in human neonates.

## 1 Introduction

Cerebral blood flow (CBF) is vital to maintaining proper cerebral perfusion and supplying the brain with necessary oxygen and metabolic substrates.^1^ Cerebral hyperemia (a higher CBF than normal) raises intracranial pressure, which may compress and damage delicate brain tissue. On the other hand, cerebral ischemia (a lower CBF than normal) may directly result in the death of brain cells. The measurement of CBF is critical for both the diagnosis and therapeutic monitoring of many cerebral vascular/cellular diseases including stroke, head trauma, and shock.^2^ Imaging of CBF with a high temporal–spatial resolution enables monitoring of fast cerebral hemodynamic changes and spatially distributed brain function.^3^^,^^4^ Moreover, acquiring data with high resolution allows for temporal–spatial averaging to improve the signal-to-noise ratio (SNR).

A variety of neuroimaging tools are used for CBF assessments; these include nuclear medicine, magnetic resonance imaging (MRI), ultrasonic techniques, and optical methods. Transcranial Doppler ultrasonography measures blood flow velocities in large vessels, which may not reflect CBF in the microvasculature. In contrast to large and expensive imaging modalities, such as nuclear medicine and MRI, optical imaging techniques based on dynamic light scattering are portable and inexpensive tools for continuous and fast measurements of cerebral hemodynamics in brain microvasculature. Near-infrared (NIR) diffuse optical technologies, such as near-infrared spectroscopy (NIRS) and diffuse correlation spectroscopy (DCS), with continuous point source illumination and photodetector detection enable noninvasive measurements of cerebral blood oxygen saturation (${\mathrm{StO}}_{2}$) and CBF, respectively, in relatively deep tissues (up to centimeters).^5^^–^^12^ However, most NIRS/DCS systems use limited numbers of discrete light sources and detectors, leading to sparse source–detector (S-D) pair measurements and a lack of combinations with the high temporal–spatial resolution and large field-of-view (FOV) needed for assessing spatially distributed cerebral hemodynamics and function.

The use of a charge-coupled device (CCD) or complementary metal-oxide-semiconductor (CMOS) camera with thousands of pixels as a two-dimensional (2D) detector array provides high-density and fast parallel sampling.^13^ For example, laser speckle contrast imaging (LSCI) with the widefield illumination and CMOS/CCD camera detection enables high-resolution 2D mapping of CBF distributions on superficial cortexes of rodents (mice and rats), although rodents’ scalps must be retracted and an invasive cranial window is needed on rats due to the limited imaging penetration depth (<1  mm).^14^^,^^15^

There have been recent advancements toward using point illumination for deep tissue penetration and CCD/CMOS camera for high-density 2D detection of diffuse laser speckle contrasts to facilitate 3D tomographic reconstruction of blood flow distributions. For example, speckle contrast optical tomography employed focused point illuminations and CCD detection of spatial diffuse speckle contrasts for 3D image of CBF distributions in tissue-simulating phantoms and rodents.^4^^,^^16^^,^^17^ We have developed an innovative speckle contrast diffuse correlation tomography (scDCT; US Patent #9/861,319, 2018^18^) method, which provides a noncontact, inexpensive, and portable tool for 3D imaging of blood flow distributions over a large FOV in relatively deep tissue volumes (up to ∼10  mm depth).^19^^–^^28^ These advanced CCD/CMOS-based techniques are inherently founded upon the same concept despite differences in nomenclature and technological evolution.

In the scDCT, a galvo mirror rapidly (switching time <1  ms) delivers continuous, coherent, point NIR light (for deep tissue penetration) to many source positions on a selected FOV with a flexible scanning pattern/density. In contrast to the NIRS and DCS systems using discrete photodetectors, scDCT uses a high-resolution scientific CMOS (sCMOS) camera with more than one million pixels as a parallel 2D detector array, which dramatically increases the sampling density and area of FOV. Boundary data from the selected S-D pairs are input to a unique finite-element-method (FEM)-based reconstruction algorithm for 3D imaging of CBF distributions.^20^^,^^21^^,^^23^^,^^24^ However, the FEM-based 3D reconstruction algorithm involves solving a forward problem to calculate light propagation through the tissue and an inverse problem to fit tissue optical properties.^10^^,^^29^ The FEM-based 3D reconstruction is complex and time consuming, taking from several minutes to hours to generate one single CBF image. Increasing the numbers of sources and detectors improves the spatial resolution with the expenses of a low temporal resolution and high computational overhead/time for 3D reconstruction, thereby prohibiting real-time applications.^29^ Moreover, 3D image reconstruction is an ill-posed inverse problem due to the nonlinear light propagation in biological tissues and limited number of S-D pairs for boundary data measurements.^30^ As such, even small errors in boundary measurements or reconstruction modeling may result in large errors in reconstructed images.^30^

To overcome these limitations, we investigated an alternative, depth-sensitive diffuse speckle contrast topography (DSCT) method for analyzing scDCT data to achieve fast and high-density 2D mapping of CBF distributions at different depths without the need for complex and time-consuming 3D reconstruction. In contrast to solving the forward and inverse problems in 3D reconstruction, DSCT simply computes blood flow indices (BFIs) for the selected S-D pairs with different distances. According to the photon diffusion theory,^31^ the penetration depth of diffusive light in biological tissues is approximately half of the S-D separation. By selecting S-D pairs with shorter or larger separations according to the imaging depth of interest, DSCT differentiates short and long photon paths through the measured tissue volume, thus generating depth-sensitive 2D maps of BFIs. Apparently, the DSCT method involves only solving forward problems with boundary data for 2D mapping, which is simpler, faster, and less sensitive to boundary measurement noises than the scDCT with 3D reconstruction. As such, a fast computation method that takes advantage of the Image Processing Toolbox™ and Parallel Computing Toolbox™ functions in MATLAB (MathWorks) was developed for rapidly calculating high-density blood flow maps.

To test the new DSCT method and compare it with other established methods, such as LSCI, a hybrid system combining scDCT and LSCI was assembled. To assess the depth sensitivity of DSCT method, new head-simulating layered phantoms with known optical/geometric properties were designed and fabricated using a 3D printing technique. Results from the DSCT and LSCI methods were compared to assess their performances. Furthermore, 2D mapping of CBF variations was performed by the DSCT in rats during ${\mathrm{CO}}_{2}$ inhalations (resulting in global CBF increases) and during unilateral and bilateral transient ligations of carotid arteries (resulting in regional CBF decreases).

## 2 Materials and Methods

This section starts with the introduction of a hybrid system combining scDCT and LSCI for comparison measurements (Sec. 2.1). Because the hardware for data acquisition in scDCT and DSCT is essentially the same, terminologies of “scDCT” and “DSCT” are used interchangeably in this paper for hardware introduction. Data analyses for the LSCI (Sec. 2.2) and new DSCT (Sec. 2.3) are then described separately. Phantom tests (Sec. 2.4) and *in-vivo* experiments in adult rats (Sec. 2.5) are followed to assess the performance of DSCT.

### 2.1 Hybrid DSCT and LSCI System

**Fig. 1:**
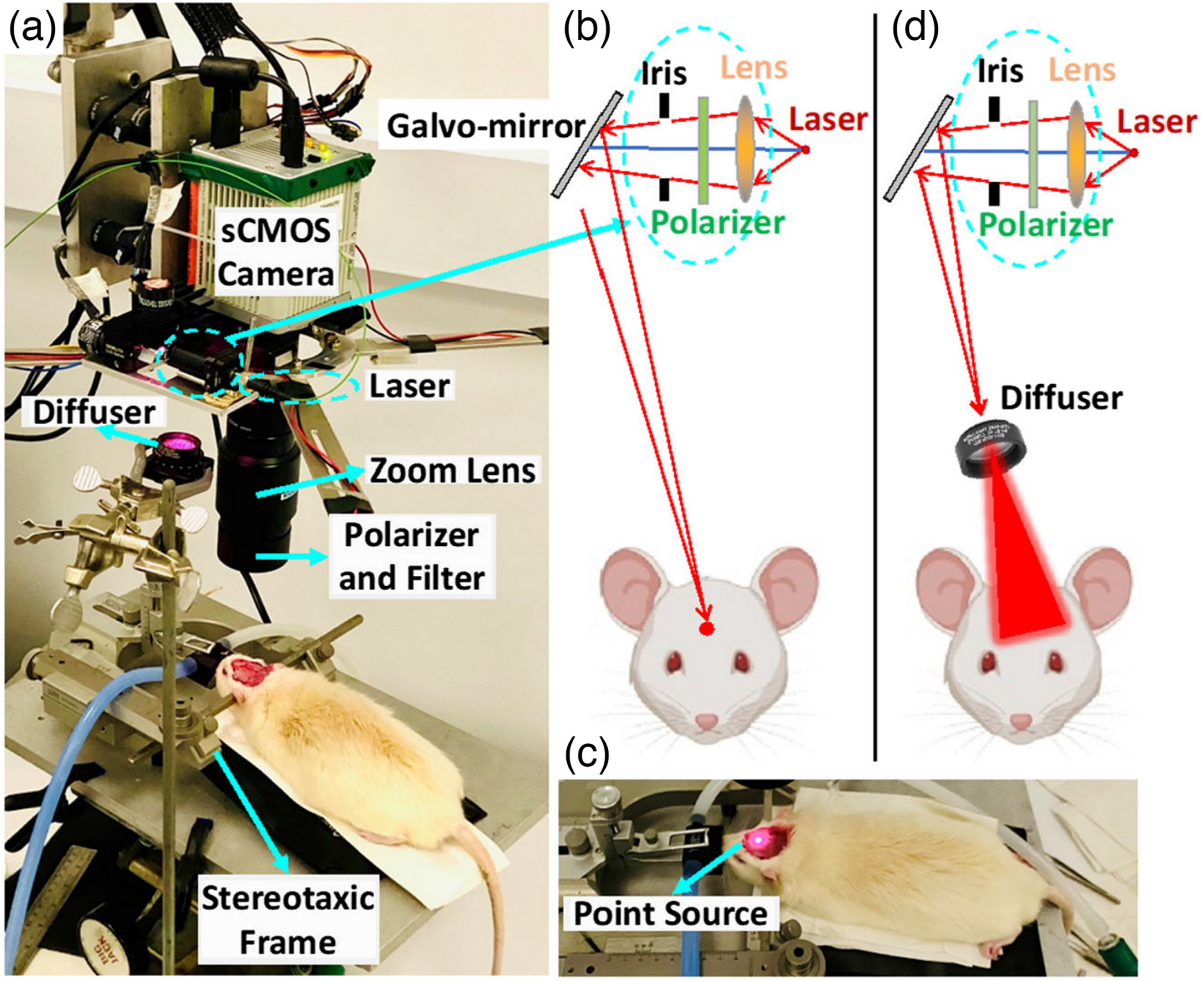
Hybrid DSCT and LSCI system for 2D mapping of CBF in rats. (a) The rat was anesthetized with 1% to 2% isoflurane and placed on a heating blanket with its head fixed on a stereotaxic frame. The hybrid instrument was set up overhead for 2D mapping of CBF distributions. (b) Optical design of DSCT. (c) NIR point source was focused on the exposed skull for DSCT measurements. (d) Optical design of LSCI. Widefield illumination was generated by manually placing a diffuser in the source path for LSCI measurements.

The DSCT acquires boundary data using the same hardware as the scDCT [Fig. 1(a)], which has been reported extensively in our previous publications.^19^^–^^25^^,^^32^^,^^33^ Briefly, a high-speed scanning galvanometer mirror positioning system (maximum scanning angle: ±12.5  deg; switching time: <1  ms; GVS002, Thorlabs) remotely and sequentially delivered coherent focused-point NIR light (780 nm, CrystaLaser) to multiple source positions on the region of interest (ROI) [Figs. 1(b) and 1(c)]. The point light was focused on the tissue surface via an achromatic lens (AC127-019-B, Thorlabs), and the light spot size was adjusted and minimized using a lever-actuated iris (SM05D5, Thorlabs). A sCMOS camera (pixels: 2048×2048; frame rate: 30/s; quantum-efficiency: 50% @800 nm; ORCA-Flash4.0, Hamamatsu Photonics) was used as a high-density 2D detector array to collect re-emitted diffuse light from the tissue for boundary measurement of spatial diffuse speckle contrasts in the selected ROI. A zoom lens (Zoom 7000, Navitar) was connected to the camera for adjusting the camera’s focus on the ROI. A high-performance long-pass filter (>750  nm; #84-761, EdmundOptics) was used to minimize the influence of ambient light on DSCT measurements.^26^ No obvious influence of ambient light was observed on the obtained raw intensity images or reconstructed BFI maps from the experiments. A pair of polarizers (LPNIRE050-B and LPNIRE200-B, Thorlabs) crossing the source and detection paths were added to reduce the specular reflection directly from the scanning light sources on the tissue surface.

For comparisons with LSCI, a hybrid DSCT and LSCI system was assembled [Fig. 1(a)]. LSCI and DSCT measurements were performed sequentially in one rat at the baseline. For LSCI measurement, an engineering diffuser (ED1-S20-MD, 20° Square Engineered Diffuser, Thorlabs) was manually placed in the source path of DSCT to generate a widefield illumination without the need for point source scanning [Fig. 1(d)]. After LSCI measurement, the diffuser was manually removed to facilitate DSCT measurement. Both DSCT and LSCI measurements utilized the same sCMOS camera with an exposure time of 2 ms. The F-number of the detection zoom lens was set as 8 to meet the Nyquist sampling rule.^14^

### 2.2 LSCI Method for 2D Mapping of Superficial CBF

The movement of light scattering particles (i.e., red blood cells) in the measured tissue volume results in spatial fluctuations of laser speckles on the tissue surface, which appear as blurred speckle patterns when recorded by the camera.^34^ Using the LSCI technique with widefield illumination [Fig. 1(d)], the raw intensity image taken from the selected ROI is converted to a spatial speckle contrast image. The spatial speckle contrast is quantified by calculating the ratio of the standard deviation (σ) of the intensity (I) to its mean intensity (⟨I⟩) in a pixel window of $N_{\text{pixels}}\times N_{\text{pixels}}$:^14^^,^^15^^,^^35^ $K_{s}=\sigma /\langle I\rangle =\sqrt{\left(\langle I^{2}\rangle -\langle I\rangle^{2}\right)}/\langle I\rangle$. The pixel window size of 3×3, 5×5, or 7×7 is usually selected to balance the detection sensitivity and spatial resolution.^35^ Larger values of $N_{\text{pixels}}$ assess spatial speckle contrasts better in terms of sensitivity at the expense of lower spatial resolutions.^14^

Although the exact relationship between $K_{s}$ and BFI is nonlinear, BFI at each pixel widow can be approximated as the inverse square of the speckle contrast: $\mathrm{BFI}∼1/K_{s}^{2}$.^34^^,^^36^ Due to its numerical simplicity, this relationship enables fast data processing of $K_{s}$ values over all pixel windows. Traditionally, two nested for-loops are utilized to calculate $K_{s}$ on each pixel window of $N_{\text{pixels}}\times N_{\text{pixels}}$. Then, a third for-loop iterates the pixel window through all pixels on the ROI to generate a 2D map of BFI.

**Fig. 2:**
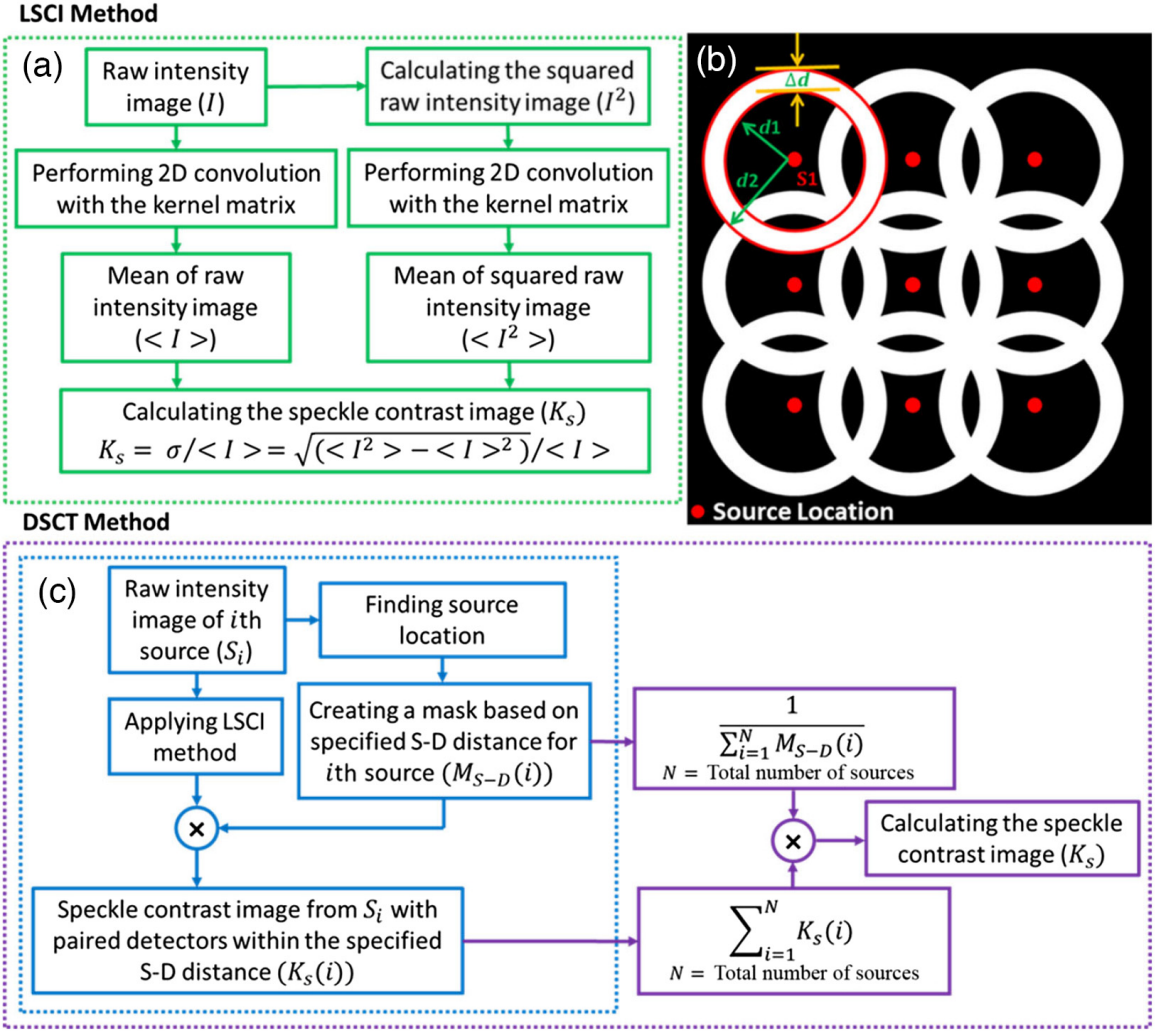
Flowchart for data processing in LSCI and DSCT methods. (a) New fast parallel computation method for LSCI data analysis. The procedures within the green dashed box were executed on each raw intensity image to calculate $K_{s}$ values. Parallel Computing Toolbox™ in MATLAB was then used to process multiple raw intensity images simultaneously and generate multiple flow maps. (b) Determination of the S-D mask in DSCT for extracting a $K_{s}$ map at the depth of d. The detectors within the specified S-D distances of [d1: d2] are selected for each source to calculate $K_{s}$ values at the depth of d. (c) New fast parallel computation method for DSCT data analysis. The procedures within the blue dashed box were executed on raw intensity images of each source ($S_{i}$) to calculate corresponding $K_{s}(i)$ values. Parallel Computing Toolbox™ in MATLAB was then used to process raw intensity images of all sources simultaneously. The steps depicted in purple boxes were implemented to reduce the artifacts arising from the summation of $K_{s}(i)$ in the overlap regions of the masks and ultimately generate one $K_{s}$ map.

We implemented a fast computation method for analyzing the LSCI and DSCT data, which takes advantage of functions from Image Processing Toolbox™ and Parallel Computing Toolbox™ in MATLAB to dynamically calculate the 2D map of BFI. Instead of using the inefficient for-loops to calculate $K_{s}$ values within the raw image, we applied MATLAB’s built-in 2D convolution function (conv2) from the Image Processing Toolbox™ to implement an implicit multi-threading approach for the fast computation.^37^ Figure 2(a) shows the steps of the new fast parallel computation method to obtain a speckle contrast image from the raw intensity image, in which the kernel matrix was defined as a square matrix of $N_{\text{pixels}}\times N_{\text{pixels}}$ with all elements equal to $1/N_{\text{pixels}}^{2}$.

Because calculations of multiple speckle contrast images are independent, MATLAB’s *parfor* control flow statement in the Parallel Computing Toolbox™ was used to execute loop iterations in parallel to compute speckle contrast images simultaneously. Specifically, in this study, MATLAB’s codes were executed on a desktop with Intel(R) Core (TM) i9-10980XE CPU @ 3.00 GHz (18-Core), NVIDIA Quadro RTX 5000 GPU. MATLAB (version: R2021a) automatically utilized all available CPU cores by default, which in this case was 18 workers, when executing parallel commands. As a result, total computation time with the new parallel computation method was reduced dramatically compared with the use of inefficient for-loops to calculate $K_{s}$ values from the raw intensity images sequentially, e.g., reducing from 6.5 h to 30 s to generate 400 flow maps with the total pixels of 2048×2048 in each map.

### 2.3 DSCT Method for 2D Mapping of CBF at Different Depths

Following our established method,^20^^,^^26^ raw intensity images were prescreened to ensure that the detected light intensities (counts) were within the linear range of our camera and above the minimal SNR requirement (i.e., SNR>3). The DSCT method first converted each of the raw intensity images taken at different source locations within the ROI into a speckle contrast image based on the fast LSCI analysis utilizing the Image Processing Toolbox™ and Parallel Computing Toolbox™ (Sec. 2.2). Because the penetration depth (d) of DSCT with point source illumination is approximately one half of the S-D distance, the Euclidean distances between each source and detectors (i.e., pixel windows of $N_{\text{pixels}}\times N_{\text{pixels}}$) were calculated. To extract a $K_{s}$ map at the depth of d, the pixels/detectors within the S-D distances of [d1:d2] from the source were averaged to improve the SNR [Fig. 2(b)]. Here, d1=2d−Δd, d2=2d+Δd, and Δd was selected empirically as 1 mm to balance the SNR and spatial resolution. More generally for each source $\left(S_{i}\right)$, a binary belt shape mask denoted as $M_{S-D}(i)$ was defined such that the pixels corresponding to the detectors located within S-D distances of [d1: d2] were assigned a value of “1,” and all other pixels were assigned a value of zero. This mask identified the paired detectors for each source to extract corresponding speckle contrast values of $K_{s}(i)$. These calculations were implemented through all sources and the resulting speckle contrast images were summed to generate one $K_{s}$ map in the selected ROI [Fig. 2(c)]. To mitigate the artifacts from the summation of $K_{s}(i)$ in the overlap regions of masks, the inverse of mask summation was multiplied by $K_{s}(i)$ summation. Note that DSCT data analyses are based on our fast parallel LSCI analysis (Sec. 2.2), thus also decreasing the total computation time dramatically compared with using inefficient for-loops methods.

The DSCT data analysis requires the input of source locations. Following our established method,^26^ the intensity image captured at each source location was analyzed to precisely determine its location. This method enabled accounting for the potential shift of each source location caused by tissue geometric factor. Briefly, the intensity image taken at each source location was first converted to a binary image. This was done by replacing all intensity values above a globally determined threshold with “1” and setting all other values to “0.” The threshold value was determined by Otsu’s method to minimize the intraclass variance of the thresholded black and white pixels.^38^ After thresholding, those pixels with a value of “1” that connected to 8 pixels in the local neighborhoods (so called eight-connected) were found and grouped together. The group with the maximum number of pixels was identified, and the rest of the groups were removed from the 2D binary image. Morphological operators, such as opening, closing, and filling, were then applied to the binary image for removing thin protrusions and isolated areas to reduce noise. Finally, the centroid and radius of the point source were determined by estimating the center of the remaining group. This automatic process was repeated for determining all source locations.^26^

### 2.4 Depth Sensitivity Tests Using New Head-Simulating Layered Phantoms

**Fig. 3:**
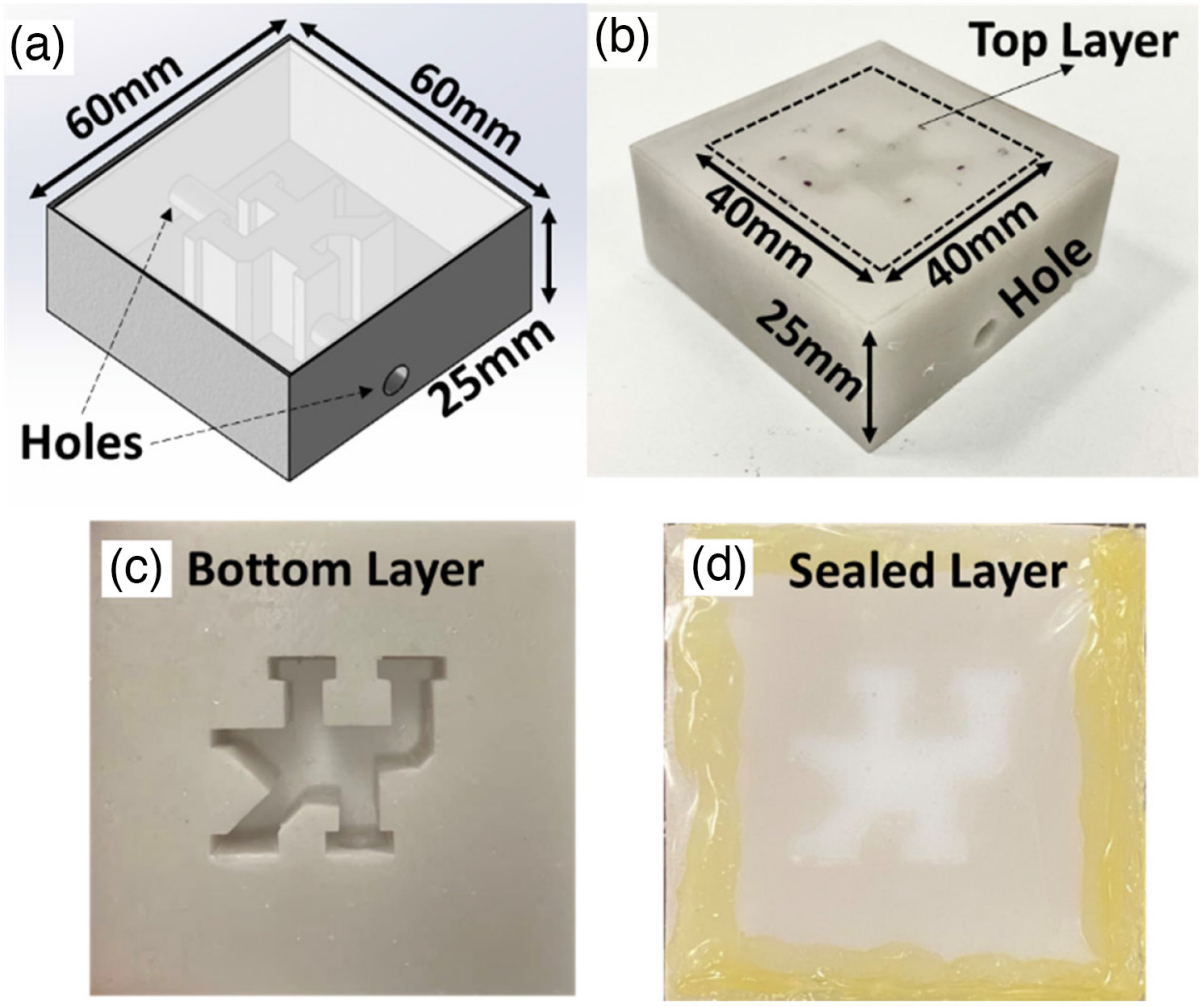
New head-simulating layered phantoms with known optical/geometry properties. (a) 3D design of solid phantom (no flow) with empty channels bearing the UK logo. The holes connected to the UK logo channels were used to fill in liquid phantoms. (b) 3D printed solid phantom with empty UK logo channels, fabricated by a 3D printer with the top layer thicknesses of 1 mm. The dashed square indicates the scanning ROI of $40\times 40\;{\mathrm{mm}}^{2}$ on the phantom surface. (c) Bottom view of solid phantom with the empty UK logo channels. (d) The empty UK logo channels were filled with liquid phantom solutions and then sealed with plastic film and a hot glue gun.

The use of tissue-simulating phantoms with known optical properties is a commonly accepted approach for the validation of new methods and technologies.^31^^,^^39^^,^^40^ In this study, head-simulating layered phantoms with known optical/geometry properties were fabricated using 3D printing techniques for testing the sensitivity of the DSCT method in mapping flow distributions at different depths. Two layers of head tissues (skull and brain) were simulated using the solid phantom with zero flow and the Intralipid liquid phantom with particle flow, respectively. Figures 3(a) and 3(b) show the 3D solid phantom (no flow) with empty channels bearing the University of Kentucky (UK) logo, fabricated by a 3D printer (SL1, Prusa). Three solid phantoms with UK logo channels were printed with the top layer thicknesses of 1, 2, and 3 mm, respectively, to mimic skulls with varied thicknesses. The solid phantoms were made of titanium dioxide (${\mathrm{TiO}}_{2}$), India ink (Black India, Massachusetts), and clear resin (eSUN Hard-Tough).^41^ The empty UK logo channels were then filled with liquid phantom solutions composed of Intralipid solution (Fresenius Kabi, Sweden), India ink, and water [Figs. 3(c) and 3(d)].^20^ India ink concentration regulates the tissue absorption coefficient ($\mu_{a}$), whereas ${\mathrm{TiO}}_{2}$ and Intralipid concentrations regulate the reduced scattering coefficient ($\mu_{s}^{\prime }$). The optical properties of both the solid and liquid phantoms were set as $\mu_{a}=0.03\;{\mathrm{cm}}^{-1}$ and $\mu_{s}^{\prime }=9\;{\mathrm{cm}}^{-1}$.^19^^,^^40^ Brownian motion of Intralipid particles inside the UK logo channels provides the particle flow to mimic the motion of red blood cells inside vessels (i.e., blood flow).^20^^,^^42^

To evaluate the impact of sampling density on spatial resolution when using DSCT, 10×10, 15×15, 20×20, and 30×30 source locations were scanned sequentially inside the ROI of $40\times 40\;{\mathrm{mm}}^{2}$ on the UK logo phantom with the top layer thickness of 1 mm. Then, the UK logo phantoms with the top layer thicknesses of 1, 2, and 3 mm were imaged by the DSCT (with 30×30 source locations) and LSCI sequentially [Fig. 3(b)]. For both DSCT and LSCI, $K_{s}$ was computed over a pixel window of 49 pixels (i.e., $N_{\text{pixels}}=7$) to balance the imaging sensitivity and spatial resolution. Five sequential flow maps over time were averaged to improve SNRs. Relative flow values were calculated by normalizing flow indices to their mean within the ROI. Finally, the results from the DSCT and LSCI were compared to demonstrate the depth sensitivity of DSCT in contrast to LSCI.

### 2.5 *In-Vivo* Experiments in Rats

To evaluate the ability of DSCT for continuous monitoring of CBF variations, adult rats with intact skulls (scalps were retracted) underwent ${\mathrm{CO}}_{2}$ inhalation, and transient carotid arterial ligations were imaged. It is well known that ${\mathrm{CO}}_{2}$ inhalation leads to global CBF increases, whereas transient arterial ligations lead to regional CBF decreases. All experimental procedures involving animals were approved by the University of Kentucky Institutional Animal Care and Use Committee (IACUC).

Six male adult Sprague–Dawley rats (age: 10 to 11 months) were imaged by the DSCT. In addition, one animal was imaged by the LSCI for comparisons. For both DSCT and LSCI, $K_{s}$ was computed over a pixel window of nine pixels (i.e., $N_{\text{pixels}}=3$) to balance the imaging sensitivity and spatial resolution. The DSCT scanned over 441 source positions (21×21) in the ROI of $20\times 20\;{\mathrm{mm}}^{2}$ on the rat head to balance temporal and spatial resolutions. 2D maps of CBF at the depths of 1 to 3 mm were generated based on the 1 to 2 mm skull thickness of adult rats.^20^ The relative time-course changes in CBF (rCBF) were calculated by normalizing BFI data to the baseline value before physiological changes.

Unlike the phantom studies in which temporal resolution is not critical for imaging constant particle flow contrasts (Sec. 2.4), a fast sampling rate is essential to capturing dynamic CBF variations caused by pathophysiological changes. Using a conventional 2D mapping method with 441 sources (21×21), the total sampling time to generate one 2D map of CBF was 60 s. In this study, a new method based on a moving window was adapted to improve the sampling rate, in which the topographic reconstruction of each CBF map was obtained when one new source position data point became available.^4^^,^^26^ As a result, the total sampling time was reduced ∼440 times (i.e., from 60 to 0.136 s).

#### 2.5.1 Continuous imaging of CBF variations in rats during ${\mathrm{CO}}_{2}$ inhalation

Each rat anesthetized with 1% to 2% isoflurane was placed on a heating blanket with its head fixed in a stereotaxic frame. After sterilizing the rat’s scalp with Betadine and 70% ethanol, a $20\times 40\;{\mathrm{mm}}^{2}$ piece of scalp was surgically removed. Following the removal, a thin layer of mineral oil was applied to the exposed skull to maintain its moisture and optical properties (Fig. 1). The noncontact DSCT probe was set above the rat head for continuous mapping of relative changes in CBF (rCBF) before (5 min), during (5 min), and after (5 min) inhalation of $92\%O_{2}/8\%{\mathrm{CO}}_{2}$. For ${\mathrm{CO}}_{2}$ inhalation, the mixed gas of $92\%O_{2}$ and $8\%{\mathrm{CO}}_{2}$ was administered through a rat nose cone.

#### 2.5.2 Continuous imaging of CBF variations in rats during transient arterial ligations

Following the ${\mathrm{CO}}_{2}$ inhalation measurement, the rat underwent transient unilateral and bilateral ligations of the common carotid artery (CCA) to create sequential decreases in rCBF at both hemispheres. For CCA ligations, hairs at the cervical surgical site were shaved and removed with hair cream, and cervical skin was disinfected with Betadine followed by 70% ethanol. A midline incision was performed to expose and isolate both the left and right CCAs. A 6-0 braided nylon suture was placed around each CCA with a loose knot. After a baseline measurement for 5 min, the right suture was tightened to ligate right CCA for 5 min. The left CCA was then also ligated to induce bilateral occlusion of CCAs for another ∼2  min. Following the release of the left CCA ligation, another 5 min of DSCT measurements were performed to record rCBF recovery by releasing the right CCA ligation as well. After the restoration of rCBF from the transient global ischemia, the rat was euthanized after ∼5  min inhalation of $100\%{\mathrm{CO}}_{2}$. One rat (rat #1) was excluded from data analysis because of a surgical complication.

### 2.6 Statistical Analysis

SPSS software (version 29) was used for statistical analysis in animal studies. The Kolmogorov–Smirnov test was performed to assess the normality of rCBF distributions. Also, repeated measures analysis of variance (ANOVA) was used to evaluate differences in rCBF variations between the different phases of stimuli (${\mathrm{CO}}_{2}$ inhalations and transient arterial occlusions) and the different hemispheres. A p-value<0.05 is considered significant for all statistical analysis.

## 3 Results

### 3.1 DSCT Performance Depends on Sampling Density

**Fig. 4:**
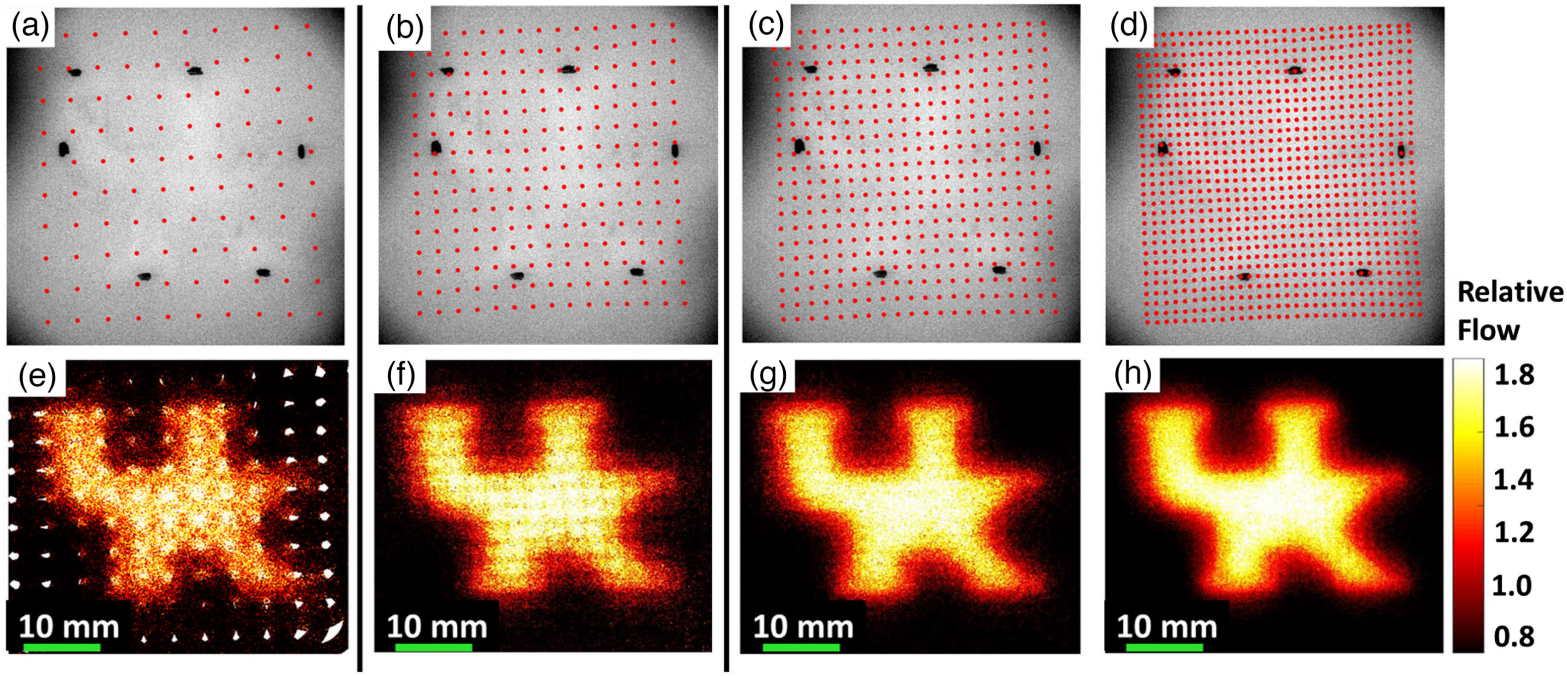
Effect of sampling density on spatial resolution of DSCT. (a)–(d) Numbers of source locations on the UK logo phantom with a top layer thickness of 1 mm: 100 (10×10), 225 (15×15), 400 (20×20), and 900 (30×30). (e)–(h) Resulting 2D flow maps at the depth of 2 mm with the source numbers of 100, 225, 400, and 900, respectively. Five sequential flow maps were averaged to improve the SNR.

Figure 4 shows the impact of sampling density on spatial resolution when using DSCT to image the UK logo phantom with the top layer thickness of 1 mm. Increasing scanning source numbers improved spatial resolution at the costs of a prolonged data acquisition time, an increased number of images for processing, and increased computation and storage demands (Table 1). More specifically, the maximum data acquisition time for obtaining one raw intensity image at one source position was ∼150  ms, including 2 ms for exposure, 33 ms for capturing one image by the camera, and 115 ms for saving data to the hard drive.

**Table 1:** Impact of sampling density on DSCT performance.

Number of sources	Total number of raw intensity images to generate one flow map	Data acquisition time (s)	Data size	Computation time for one depth (s)
10 × 10	100	15	800 MB	12
15 × 15	225	34	1.75 GB	20
20 × 20	400	60	3.12 GB	30
30 × 30	900	132	7.04 GB	85

### 3.2 DSCT Enables 2D Mapping of Phantom Flow Contrasts with Depth Sensitivity

**Fig. 5:**
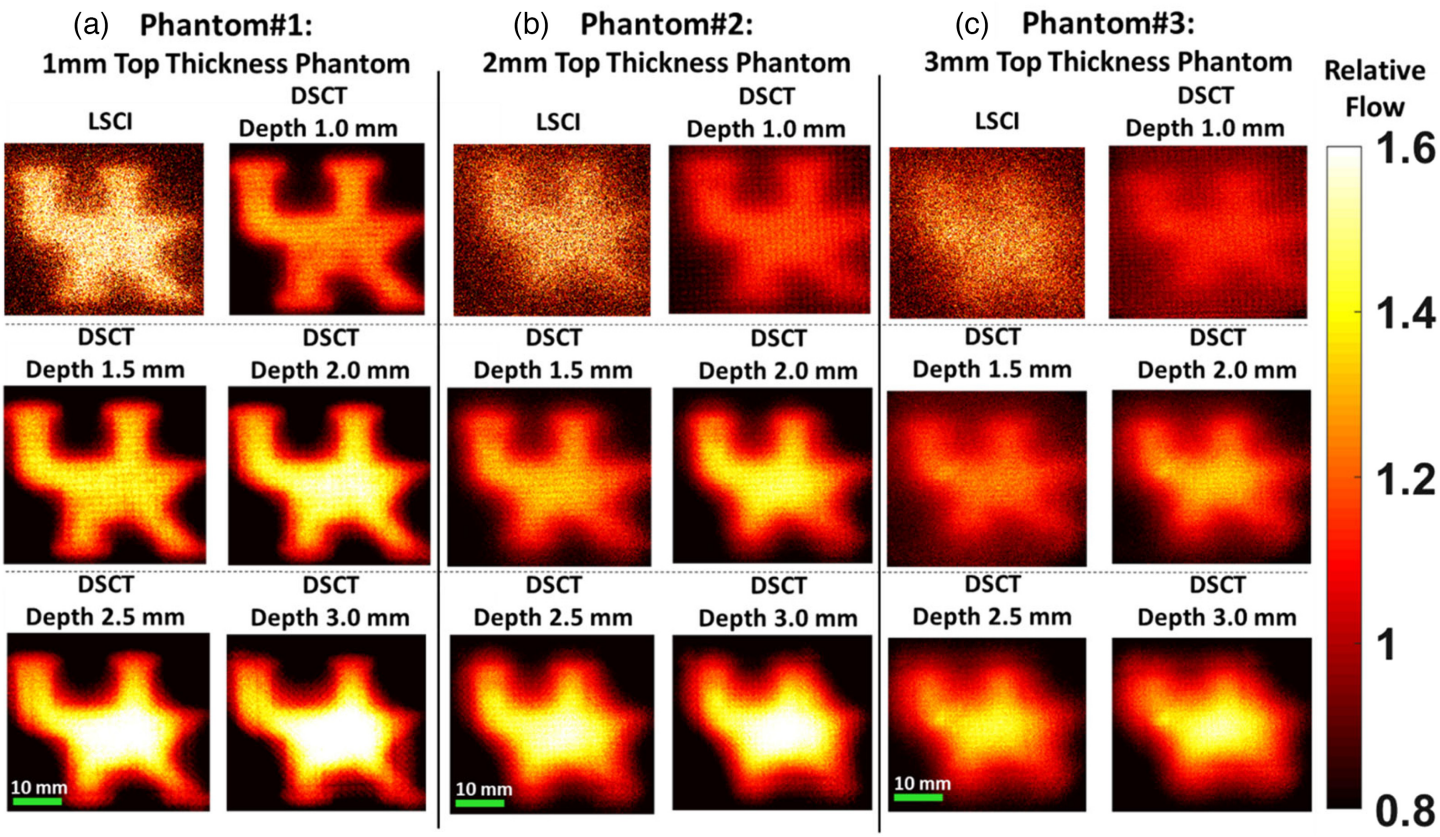
DSCT and LSCI measurement results for UK logo phantoms. (a), (b) Resulting 2D maps of Intralipid particle flow contrasts on three phantoms with the top layer thicknesses of 1, 2, and 3 mm, respectively. Flow indices were normalized to their mean values to generate relative flow maps for comparisons.

Figure 5 shows the results using the DSCT and LSCI to image UK logo phantoms with top layer thicknesses of 1, 2, and 3 mm, respectively. For DSCT measurements, 30×30 source locations were scanned over the selected ROI of $40\times 40\;{\mathrm{mm}}^{2}$ on the phantoms. Higher flow contrasts were observed at deeper depths with larger S-D separations, indicating the depth sensitivity of the DSCT method. SNRs decreased with the increases of imaging depth and top layer thickness (i.e., from 1 to 3 mm). These results are expected as the deeper penetration and thicker top layer resulted in fewer diffused photons being detected, thus leading to lower SNRs. By contrast, SNRs and spatial resolutions of superficial LSCI measurements were much worse than those of DSCT measurements, especially on the phantoms with thicker top layers.

### 3.3 DSCT Enables 2D Mapping of rCBF at Different Depths in Rats

**Fig. 6:**
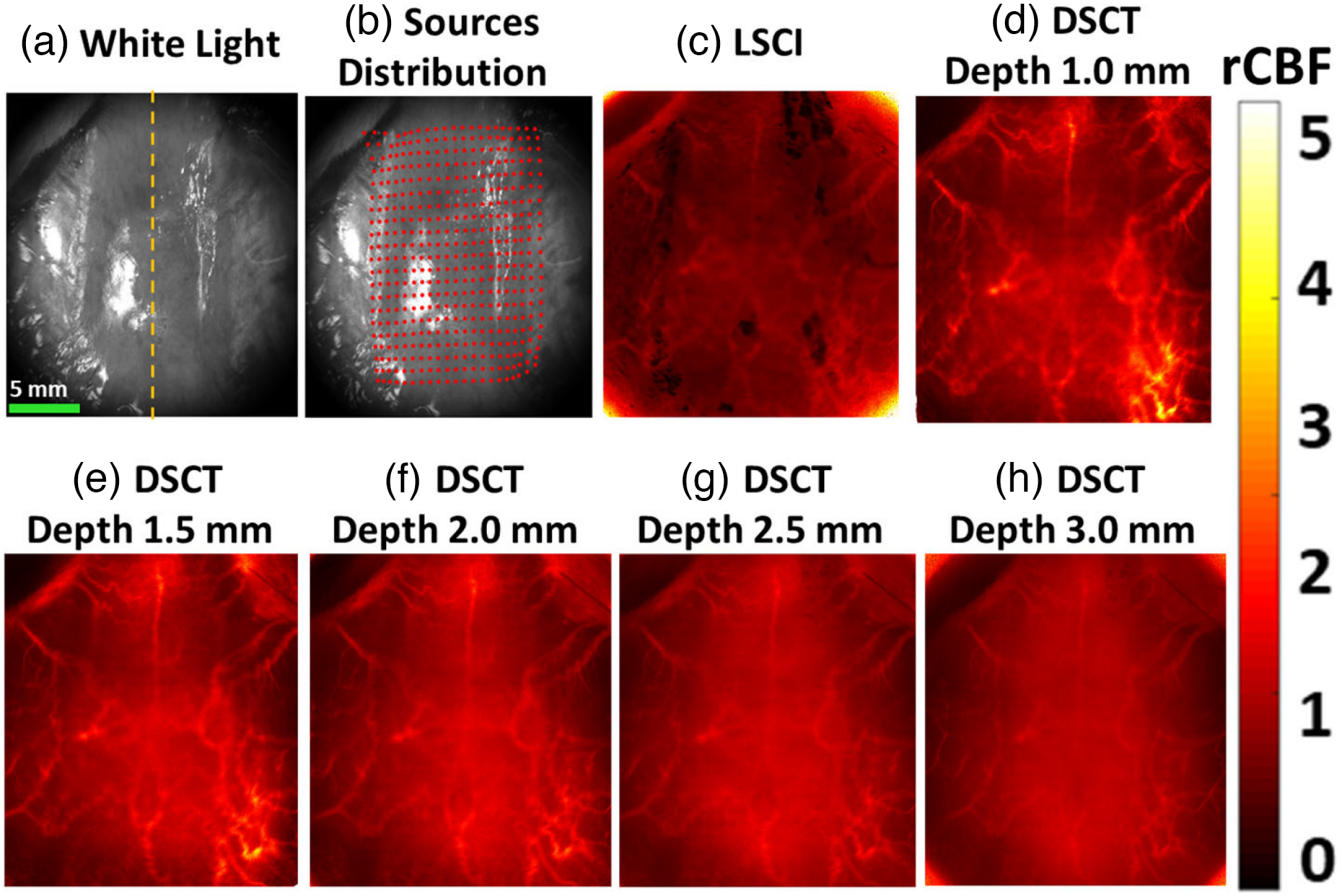
2D mapping of rCBF distributions with LSCI and DSCT. (a) A white light image of a rat head with nose up (rat #4, scalp was retracted). The yellow dotted line indicates the central line that separates the left and right hemispheres (RH). (b) Sources distribution (red dots) on the ROI of $20\times 20\;{\mathrm{mm}}^{2}$. (c) 2D map of rCBF, imaged by LSCI. (d)–(h) 2D maps of rCBF at different depths from 1.5 to 3.0 mm, imaged by DSCT.

Figures 6(a) and 6(b) show white light images of a rat head (scalp was removed) and 441 source positions (21×21) on the rat skull for DSCT measurements. Figure 6(c) shows rCBF map of a representative rat (rat #4), imaged by the LSCI. Figures 6(d)–6(h) show rCBF maps of rat #4 at depths varying from 1 to 3 mm, imaged by the DSCT. As observed in phantom measurements (Sec. 3.2), DSCT enables 2D mapping of rCBF distributions at various depths, with higher spatial resolutions and SNRs than LSCI.

### 3.4 DSCT Captures Dynamic rCBF Responses During ${\mathrm{CO}}_{2}$ Inhalations in Rats

**Fig. 7:**
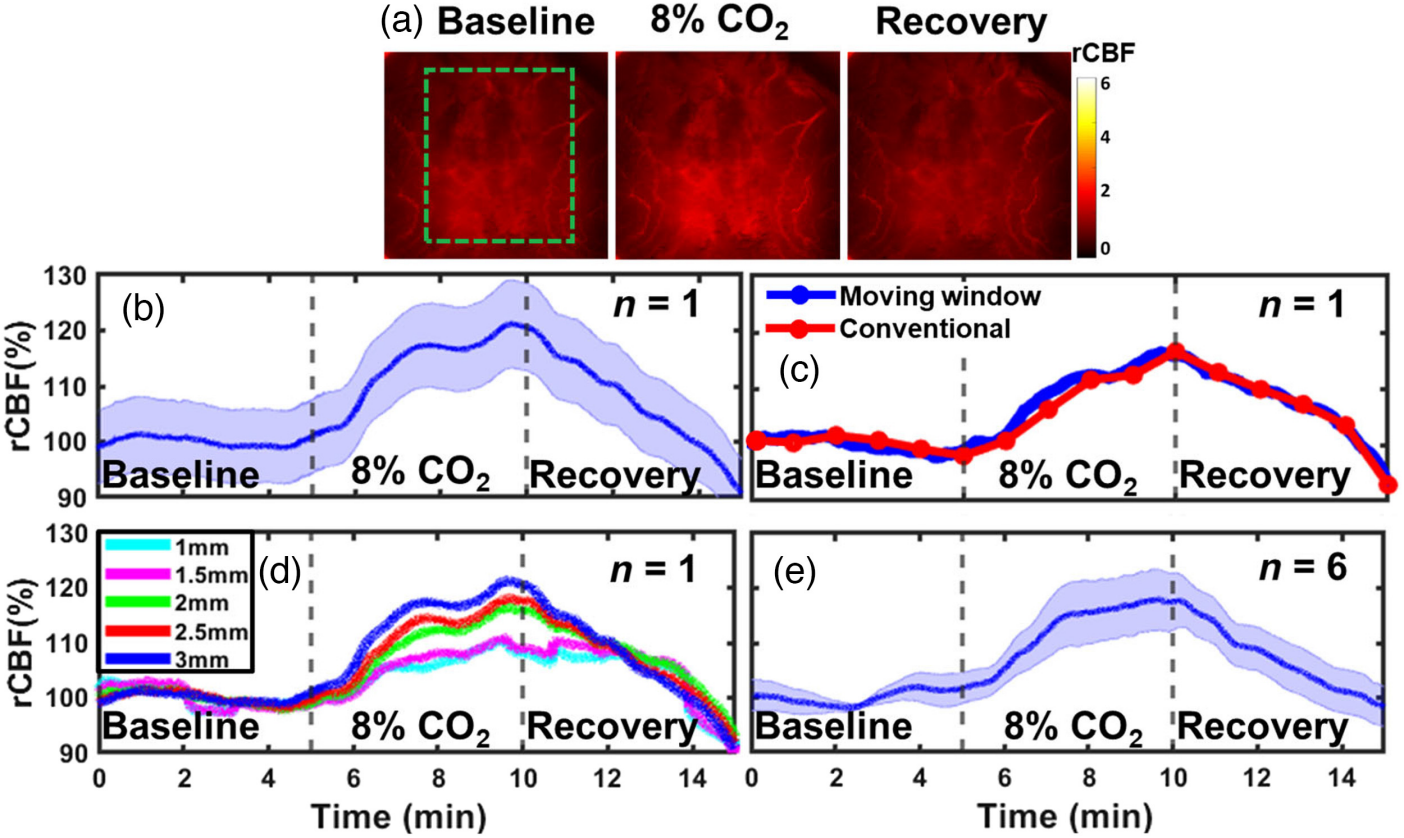
DSCT measurements of rCBF responses to ${\mathrm{CO}}_{2}$ inhalation in rats. (a) 2D maps of rCBF at the depth of 3 mm before, during, and after $8\%{\mathrm{CO}}_{2}$ inhalation in an illustrative rat (rat #2). The dashed green box indicates the selected brain region for data analysis. (b) Time-course changes in rCBF at a depth of 3 mm, averaged over the brain region (dashed green box) and reported as mean value ± standard deviation in one rat (n=1, rat #2). The shaded area represents standard deviations of spatial changes. (c) Comparison of time-course changes in rCBF (mean values) between the conventional reconstruction (red curve) and moving window (blue curve) methods (rat #2). (d) Comparison of time-course changes in rCBF (mean values) in one rat (n=1, rat #2) at different depths ranging from 1.0 to 3.0 mm (moving window method). (e) Average time-course rCBF changes (moving window method) at a depth of 3 mm over six rats (mean value ± standard error). The shaded area represents standard errors.

Figure 7(a) shows typical blood flow maps in a representative rat (rat #2) at the depth of 3.0 mm before, during, and after $8\%{\mathrm{CO}}_{2}$ inhalation, measured by the DSCT with 441 source positions (21×21). Figure 7(b) shows time-course changes in rCBF in one rat (rat #2), calculated by the moving window method and normalizing BFI data to their baseline values (assigned as “100%”) before ${\mathrm{CO}}_{2}$ inhalation. Figure 7(c) compares the results of time-course rCBF changes between the conventional reconstruction (red curve) and moving window (blue curve) methods. As expected, both methods generated similar trends in rCBF, although the sampling rate of the moving window method was much higher than the standard reconstruction method. Figure 7(d) compares time-course changes in rCBF at different depths ranging from 1.0 to 3.0 mm. As expected, rCBF changes in deeper brain tissues (depth≥2.0  mm) were higher than those in the skull (depth≤1.5  mm). Figure 7(e) shows average time-course changes in rCBF (moving window method) over six rats. Table 2 and Table S1 in the Supplemental Material summarize the average changes in rCBF and corresponding significance levels (p-values), respectively, during different phases of ${\mathrm{CO}}_{2}$ inhalation. The $8\%{\mathrm{CO}}_{2}$ led to a significant increase in rCBF at the endpoint of inhalation compared with the baseline (mean ± standard error: 117%±5%; repeated measures ANOVA: p-value=0.024), which agrees with previously reported changes in rCBF.^20^ There was also a significant difference (repeated measures ANOVA: p-value=0.046) between the $8\%{\mathrm{CO}}_{2}$ inhalation and the endpoint of the recovery phase.

**Table 2:** Average rCBF variations (mean ± standard error) from their baselines during ${\mathrm{CO}}_{2}$ inhalation over six rats (moving window method).

	Baseline	8% ${\mathrm{CO}}_{2}$ inhalation	Recovery
**Brain region**	100% ± 0.2%	117% ± 5%	107% ± 8.5%

### 3.5 DSCT Captures Dynamic rCBF Responses to Transient Arterial Ischemia and Hypoxia in Rats

**Fig. 8:**
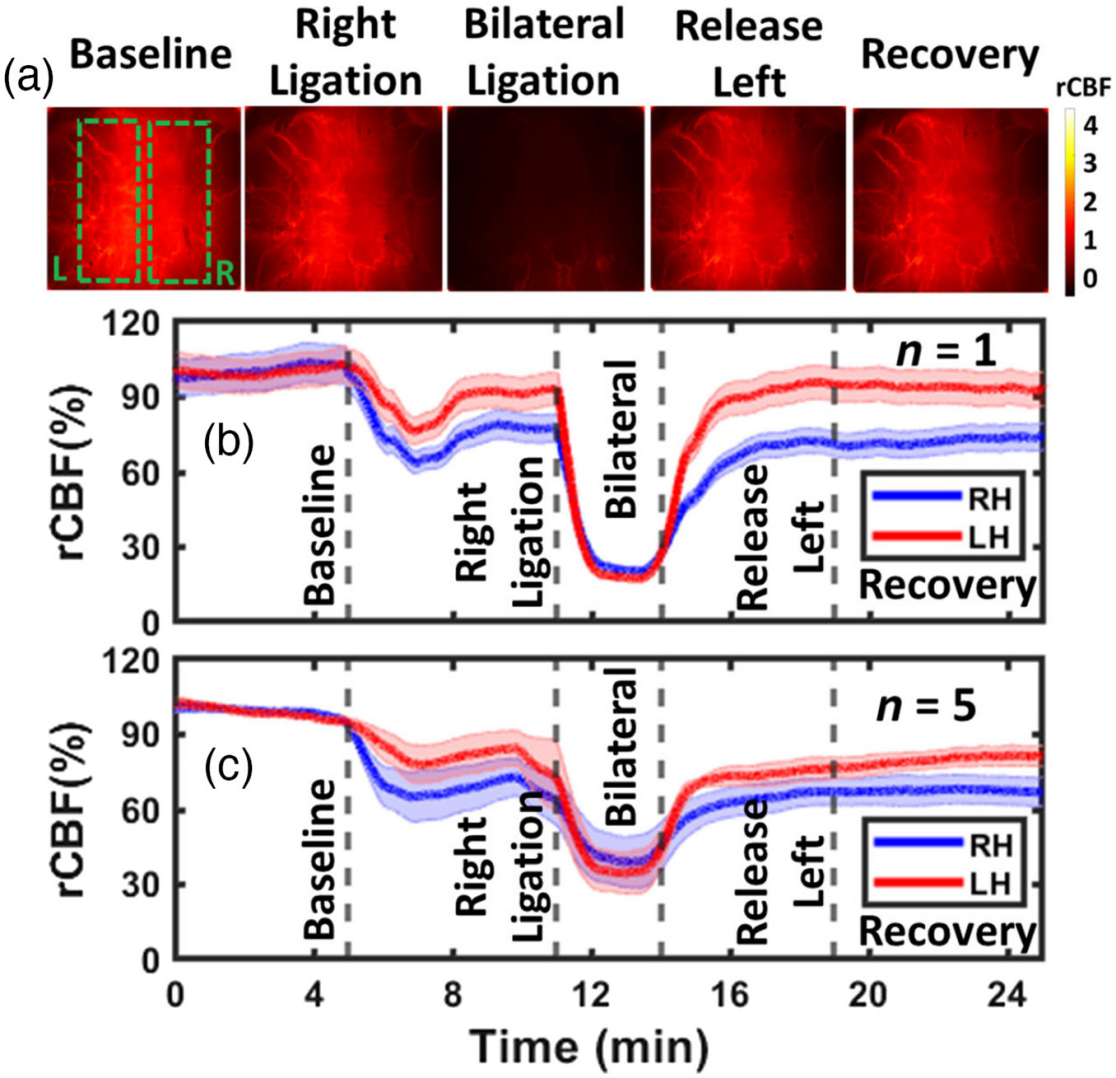
DSCT measurements of rCBF responses to sequential unilateral and bilateral CCA ligations in rats. (a) 2D maps of rCBF at the depth of 3 mm before, during, and after unilateral and bilateral CCA ligations in an illustrative rat (rat #3). The dashed green boxes indicate selected brain regions at RH and LH for data analyses. (b) Time-course changes in rCBF (moving window method), averaged over the brain regions (dashed green boxes) and reported as mean value ± standard deviation (rat #3). The shaded area represents standard deviations. (c) Average time-course rCBF changes over five rats (mean value ± standard error). The shaded area represents standard errors.

Figure 8(a) shows typical blood flow maps in a representative rat (rat #3) at the depth of 3.0 mm before, during, and after sequential CCA ligations, measured by the DSCT with 441 source positions (21×21). Figure 8(b) shows time-course changes in rCBF at the right hemisphere (RH) and left hemisphere (LH) of one rat (rat #3), which were calculated by the moving window method and normalizing BFI data to the baseline values prior to CCA ligations. Figure 8(c) shows average time-course changes in rCBF over five rats. Table 3 and Table S2 in the Supplemental Material summarize the average changes in rCBF and corresponding significance levels (p-values), respectively, during different phases of CCA ligations. As expected, the sequential right and left CCA ligations as well as subsequent releasing resulted in significant alterations in rCBF in both hemispheres compared with their baselines (repeated measures ANOVA: p-value<0.05). In particular, the bilateral ligation led to significant reductions in rCBF in both hemispheres, compared with all other phases of transient CCA ligations (repeated measures ANOVA: p-value<0.05). These results agree with our previous study in adult rats utilizing scDCT with 3D reconstruction.^20^

**Table 3:** Average rCBF variations (mean ± standard error) from their baselines during unilateral and bilateral CCA ligations over five rats (moving window method).

	Right ligation (%)	Bilateral ligation (%)	Release left ligation (%)	Release right ligation (recovery) (%)
**RH**	67% ± 9	41% ± 10	63% ± 6	68% ± 6
**LH**	78% ± 9	37% ± 9	72% ± 4	80% ± 4

**Fig. 9:**
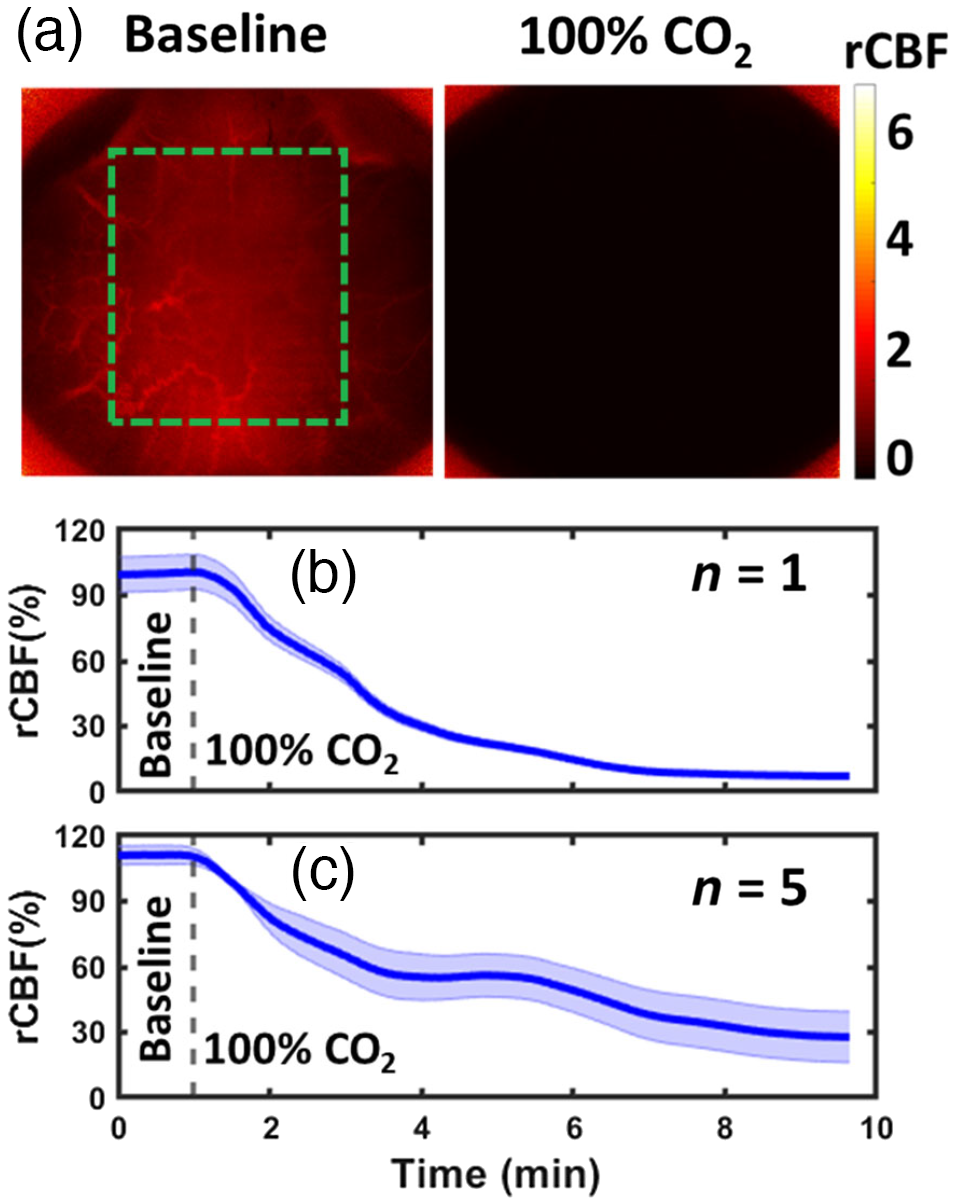
DSCT measurements of rCBF variations during asphyxia ($100\%{\mathrm{CO}}_{2}$) in rats. (a) 2D maps of rCBF at the depth of 3 mm before and at the end of asphyxia challenge in an illustrative rat (rat #4). The dashed green box indicates the selected brain region for data analysis. (b) Time-course changes in rCBF, averaged over the brain region (dashed green boxes) and reported as mean value ± standard deviation. The shaded area represents standard deviations. (c) Average time-course rCBF changes over five rats (mean value ± standard error). The shaded area represents standard errors.

Figure 9(a) shows typical blood flow maps in a representative rat (rat #4) at the depth of 3.0 mm before and during $100\%{\mathrm{CO}}_{2}$ euthanasia, measured by the DSCT with 441 source positions (21×21). Figure 9(b) shows time-course changes in rCBF, which were calculated by the moving window method and normalizing BFI data to the baseline values prior to asphyxia. Figure 9(c) shows average time-course changes in rCBF over five rats. At the end, rCBF values were close to “zero,” meeting the physiological expectation.

## 4 Discussion and Conclusions

The assessment of cerebral hemodynamics is crucial for the diagnosis and management of cerebral vascular diseases. The recent development of an innovative noncontact scDCT in our laboratory provides a low-cost and portable tool for 3D imaging of CBF distributions with an adjustable FOV and S-D configuration.^20^^,^^21^^,^^23^^,^^24^ The spatial resolution of scDCT depends on the numbers of scanning coherence sources and detectors/pixels on the sCMOS camera. However, higher numbers of sources and detectors result in a longer computational time required for 3D reconstruction and a lower temporal resolution. To reduce the computation time, we present a new DSCT method with parallel computation for analyzing scDCT data to achieve fast and high-density 2D mapping of flow distributions at different depths without the need for complex and time-consuming 3D reconstruction (Fig. 1).

Specifically, DSCT takes advantages of Image Processing Toolbox™ and Parallel Computing Toolbox™ functions in MATLAB to rapidly generate a 2D map of CBF (Fig. 2). To achieve fast calculation of $K_{s}$ values from raw intensity images, MATLAB’s built-in 2D convolution function was used from the Image Processing Toolbox™. Because raw intensity images obtained at different source positions are independent, multiple 2D speckle contrast images at different depths (by selecting different S-D separations) are computed and generated simultaneously by executing parallel loop iterations using MATLAB’s Parallel Computing Toolbox™. The use of parallel computing with high performance CPUs reduced the processing time significantly compared with the inefficient for-loops method. For instance, the time required to generate 400 flow maps (each with total pixels of 2048×2048) decreased dramatically from 6.5 h to 30 s.

To tackle low temporal resolution during high density scanning for capturing dynamic rCBF variations, a moving window method in which a new 2D CBF map was produced as soon as the raw image from one new source position data became available was employed to increase the temporal resolution of DSCT.^4^^,^^26^ For example, the moving window method with 441 sources (21×21) updated the data at a new source position every 0.136 s. As a result, the sampling rate of the moving window method (7.35 Hz) was ∼440 times higher than the conventional reconstruction method (0.017 Hz). It should be noted that the moving window method increased the temporal resolution significantly at the expense of only utilizing data from one updated source location to generate a new image. Nevertheless, we were able to measure rapid changes in rCBF during ${\mathrm{CO}}_{2}$ and CCA ligation tests. It is possible to further increase the sampling rate by optimizing the number of sources and selecting high-quality cameras with faster frame rates. Also, our DSCT with a fast and high-density sampling holds the potential for use in mapping brain functional connectivity,^43^ which is a subject of our future study.

To evaluate the effect of sampling density on spatial resolution and depth sensitivity of the DSCT method for 2D flow mapping, new head-simulating layered phantoms with the UK logo were fabricated by a 3D printing technique (Fig. 3). The phantoms were printed with a top solid layer to mimic the skull (no flow) and the UK logo filled by a liquid phantom solution (with Intralipid particle flow). Higher sampling density improves the spatial resolution at the expense of a longer data acquisition time (Fig. 4 and Table 1). Therefore, it is necessary to balance the spatial and temporal resolutions for specific applications. Results from sequential DSCT and LSCI measurements demonstrate that DSCT enables 2D mapping of the particle flow at different depths through the top layer (skull) with varied thicknesses of 1 to 3 mm (Fig. 5). We note that relative flow maps were generated by normalizing the measured flow indices to their mean values within the ROI, obtained from both solid and liquid phantoms. This normalization somewhat reduces the flow contrast between the solid and liquid phantoms, leading to an underestimation of flow contrasts. By contrast, LSCI can barely map the UK logo phantoms with top layer thickness of ≥1 mm.

Based on phantom test results, we optimized DSCT’s setup for *in-vivo* animal experiments. We tested the ability of DSCT for continuous monitoring of temporal and spatial variations in rCBF by imaging global rCBF increases during ${\mathrm{CO}}_{2}$ inhalations and regional rCBF decreases during CCA ligations in adult rats. Approximately 3 min after $8\%{\mathrm{CO}}_{2}$ inhalation, rCBF increased globally and significantly: 117%±5% of its baseline (Fig. 7, Table 2, and Table S1 in the Supplemental Material). These results are consistent with our previous study on adult rats utilizing the scDCT with 3D reconstruction (119%±8% during 10% ${\mathrm{CO}}_{2}$ inhalation).^20^ Bilateral CCA ligations resulted in significant reductions in rCBF: 41%±10% and 37%±9% of their baselines in the RH and LH, respectively [Fig. 8(c), Table 3, and Table S2 in the Supplemental Material]. rCBF variations during bilateral CCA ligations agree with our previous study in adult rats utilizing scDCT with 3D reconstruction (32%±11% and 34%±10%).^20^ These results suggest that both DSCT and scDCT enable the detection of global and regional rCBF changes in deep brains. However, scDCT employs limited numbers of sources and detectors (e.g., 5×5 sources and 21×21 detectors) for a complex and time-consuming 3D reconstruction, whereas DSCT utilizes large numbers of sources and detectors (e.g., 21×21 sources and 2048×2048 detectors) to achieve fast and high-density 2D mapping of CBF distributions at different depths.

Real-time or nearly real-time mapping and reporting of CBF by the DSCT offer a range of benefits. Researchers can conduct experiments with faster turnaround times, thus enabling iterative testing and optimization, which is crucial for refining the experimental setup and methodology. Real time is particularly beneficial when immediate feedback is needed for making decisions, such as during a surgical procedure or medical intervention. Efficient data analysis capability is essential for reporting rapid physiological responses or transient events with a high sampling rate and large data size. Although this study focuses on testing DSCT in animals, the ultimate goal is to translate this technology to clinical settings in which real-time monitoring/feedback is essential.

In conclusion, we presented a depth-sensitive DSCT method for rapid 2D mapping of blood flow distributions in deep brains. The performance of DSCT was evaluated in head-simulating tissue phantoms and *in-vivo* experiments in adult rats. The results from DSCT agreed with those reported previously using scDCT in adult rats with similar experimental protocols. Because the hardware for data acquisition in the scDCT and DSCT is essentially the same, they share the same advanced features, including noncontact hardware, flexible S-D configuration, adjustable ROI/FOV, and a cost-effective instrument. Compared with the scDCT technique with 3D reconstruction, the DSCT method with a fast sampling rate and parallel computation enables fast and high-density 2D mapping of blood flow distributions at different depths. With more optimizations, we expect to ultimately offer a noninvasive, noncontact, fast, high resolution, portable, and inexpensive brain imager for basic neuroscience research in small animal models and for translational studies in human neonates.

## Data Availability

All data supporting the findings of this study are available from the corresponding author upon request.
