# Supplementary material for: Depth-sensitive diffuse speckle contrast topography for high-density mapping of cerebral blood flow in rodents
Source: Neurophotonics. 2023 Nov 14;10(4):045007. doi: 10.1117/1.NPh.10.4.045007 (PMC10704187; doi:10.1117/1.NPh.10.4.045007)
Supplement: Supplementary file 1 [file NPh_010_045007_SD001.pdf]

Supplemental Material

Table S1 shows significance levels (p-values) of changes in rCBF during different phases of 8%CO_2 inhalation using repeated measures ANOVA. The 8%CO_2 led to a significant increase in rCBF at the endpoint of inhalation compared to the baseline (repeated measures ANOVA: p-value = 0.024). There was also a significant difference (repeated measures ANOVA: p-value = 0.046) between the 8%CO2 inhalation and the endpoint of recovery phase.

Table S1: Repeated measures ANOVA to assess differences in rCBF variations between different phases of CO_2 inhalation

		p-value
**Baseline**	**8%CO_2 Inhalation**	0.024*
	**Recovery**	0.733
**8% CO_2 Inhalation**	**Baseline**	0.024*
	**Recovery**	0.046*
**Recovery**	**Baseline**	0.733
	**8% CO_2 Inhalation**	0.046*

*The mean difference is significant at the 0.05 level.

Table S2 shows significance levels (p-values) of changes in rCBF during different phases of CCA ligations using repeated measures ANOVA. As expected, the sequential right and left CCA ligations as well as subsequent releasing resulted in significant alterations in rCBF in both hemispheres compared to their baselines (repeated measures ANOVA: p-value < 0.05). Particularly, the bilateral ligation led to significant reductions in rCBF in both hemispheres, compared to all other phases of transient CCA ligations (repeated measures ANOVA: p-value < 0.05).

Table S2: Repeated measures ANOVA to assess differences in rCBF variations between different phases of ligation in right hemisphere (RH) and left hemisphere (LH)

		Right Hemisphere (RH) p-value	Left Hemisphere (LH) p-value
**Baseline**	Right Ligation	0. 003*	0.029*
	Bilateral Ligation	<0.001*	<0.001*
	Release Left Ligation	<0.001*	<0.001*
	Release Right Ligation (Recovery)	<0.001*	0.005*

**Right Ligation**	Baseline	0.003*	0.029*
	Bilateral Ligation	0.005*	<0.001*
	Release Left Ligation	0.482	0.291
	Release Right Ligation (Recovery)	0.925	0.780
**Bilateral Ligation**	Baseline	<0.001*	<0.001*
	Right Ligation	0.005*	<0.001*
	Release Left Ligation	0.035*	0.003*
	Release Right Ligation (Recovery)	0.028*	0.002*
**Release Left Ligation**	Baseline	<0.001*	<0.001*
	Right Ligation	0.482	0.291
	Bilateral Ligation	0.035*	0.003*
	Release Right Ligation (Recovery)	0.060	0.005*
**Release Right Ligation (Recovery)**	Baseline	<0.001*	0.005*
	Right Ligation	0.925	0.780
	Bilateral Ligation	0.028*	0.002*
	Release Left Ligation	0.060	0.005*

*The mean difference is significant at the 0.05 level.
